# Impact of respiratory training combined with electrical phrenic nerve stimulation on pulmonary and trunk function in individuals who have recently experienced a stroke

**DOI:** 10.3389/fneur.2025.1579421

**Published:** 2025-06-17

**Authors:** Yan-Fang Sui, Zhen-Hua Song, Jing-Qin Shi, Shan-Shan Wang, Bin-Bin Li, Liang-Qian Tong

**Affiliations:** ^1^Department of Rehabilitation Medicine, Affiliated Haikou Hospital of Xiangya Medical College, Central South University, Haikou, China; ^2^Department of Nuclear Medicine, Affiliated Haikou Hospital of Xiangya Medical College, Central South University, Haikou, China

**Keywords:** breathing training, electrical phrenic nerve stimulation, pulmonary function, stroke, trunk function

## Abstract

**Objective:**

This study aims to explore the effects of combining phrenic nerve electrical stimulation with respiratory training on pulmonary and trunk function in post-stroke individuals. Rationale for combining these interventions stems from the diaphragm’s dual role in respiration and postural control, as well as the limitations of conventional respiratory training in addressing phrenic nerve dysfunction and impaired diaphragm coordination after stroke.

**Methods:**

In this single-blinded randomized controlled trial, 160 early stroke patients were randomly assigned via computer-generated random number tables with allocation concealment using sealed opaque envelopes to a control group receiving standard therapy and an experimental group receiving additional phrenic nerve stimulation and breathing training. Each group comprised 80 patients. To evaluate the trunk function and balance before and after the treatment, the Sheikh Trunk Control Scale, Berg Balance Scale (BBS), and Balance Feedback Training Device were utilized. Additionally, pulmonary function was assessed using a pulmonary function measuring instrument.

**Results:**

Following 4 weeks of treatment, there was a statistically significant enhancement in the Sheikh Trunk Control Scale and BBS scores, respiratory muscle strength index, and peak inspiratory flow rate for patients in both groups (*p* < 0.05). Additionally, there were significant reductions in measures related to balance, including movement length, movement area, as well as mean anterior–posterior and left–right movement speeds (*p* < 0.05). Consequently, after the 4-week treatment period, the trunk function and balance, pulmonary function all improved in the experimental group.

**Conclusion:**

Combining phrenic nerve stimulation with respiratory training can effectively improve lung and core functions during post-stroke rehabilitation. However, generalizability is limited by the short follow-up period and strict exclusion criteria. Future research should explore long-term outcomes and compare combined interventions with standalone therapies.

## 1 Introduction

Stroke stands as a prominent contributor to disability on a global scale. According to the World Health Organization (WHO), stroke is the second leading cause of death and the third leading cause of disability-adjusted life years (DALYs) worldwide, with an estimated 12.2 million new cases annually. In addition to the data from China, which reveals a concerning trend in stroke prevalence (from 1.89% in 2012 to 2.58% in 2019 among individuals aged 40 years and above), global statistics underscore the urgent need for effective rehabilitation strategies (1). For instance, in the United States, stroke affects nearly 800,000 people each year, with approximately 7 million stroke survivors living with long-term disabilities (2). Similarly, in Europe, stroke accounts for over 1.1 million deaths annually, with significant economic and social burdens due to disability and reduced quality of life (3). In 2019, the number of individuals aged 40 years and above who either suffered from or had experienced a stroke reached approximately 17.04 million (4).

It is important to note that stroke not only results in motor dysfunction but also neuromuscular respiratory muscle weakness, which significantly contributes to respiratory impairment. This respiratory decline and reduced ability to cough effectively among stroke patients heighten their susceptibility to lung infections, which in turn prolong hospital stays and increase the economic and social burdens associated with stroke care (5). Consequently, early pulmonary rehabilitation plays a vital role in the recovery of stroke patients (6–9). Nevertheless, current rehabilitation approaches for early stroke (which is defined as the time from 48 h of stable disease to 3 weeks after onset) patients predominantly target limb functionality, often overlooking the crucial aspect of pulmonary rehabilitation (10). Traditional respiratory training alone may inadequately address diaphragm dysfunction, a critical factor in post-stroke respiratory and postural impairments. Traditional respiratory training alone primarily focuses on inspiratory muscle strengthening (e.g., diaphragmatic breathing) but fails to address phrenic nerve dysfunction or impaired diaphragm coordination in stroke patients, which are critical contributors to respiratory and postural deficits.

Trunk stability encompasses both core stability and postural control. Core muscles, when engaged, regulate the pressure in the thoracic and abdominal areas by contracting the diaphragm. This action enhances the strength of the pelvic and spinal regions, contributing to overall bodily stability (11). Furthermore, the diaphragm, aside from its role in respiration, plays a biomechanical role in posture by altering muscle length and structure when it interfaces with the spine (12). Hence, it is clear that the diaphragm is not solely responsible for breathing but also plays a crucial part in maintaining posture. In stroke patients, respiratory issues often coincide with impaired diaphragmatic function, undermining its role in postural control and significantly increasing the risk of balance problems.

Therefore, strengthening diaphragmatic function through training and breathing exercises can aid in the recovery of trunk function.

In recent times, electrical phrenic nerve stimulation has gained recognition in pulmonary rehabilitation for conditions like chronic obstructive pulmonary disease, spinal cord injury, chronic heart failure, and sleep disorders. Phrenic nerve stimulation can reduce pulmonary artery pressure, and the specific effects can be reflected in the reduction of PaCO^2^, the increase of 6-min walking distance and the promotion of expectoration (13). However, its application for stroke patients has been relatively limited. Electrical phrenic nerve stimulation bypasses these central deficits by directly activating the nerve, enhancing neuromuscular transmission and promoting neuroplasticity in residual pathways. Therefore, this study delves into the importance of combining electrical phrenic nerve stimulation with breathing training to enhance the recovery of pulmonary and trunk functions in early stroke patients.

## 2 Participants and methods

### 2.1 Participants

In this study, 160 patients suffering from motor impairment following a stroke who were admitted to the hospital between September 1, 2020, and December 31, 2022, were enrolled and randomly divided into two groups, with 80 patients in each group and no patients dropped out. This RCT utilized a convenience sample, with participants recruited based on availability, accessibility, and willingness to participate. After enrollment, participants were randomly assigned to either the intervention or control group using a computer-generated random number table. Allocation concealment was maintained through sealed opaque envelopes. Assessors and physicians providing treatments were blinded to group allocation. Baseline characteristics, including age, gender, and disease duration, were compared to ensure no significant differences between the groups. The sample size was calculated based on a power analysis with an assumed effect size of 0.5, a power of 0.8, and a significance level of 0.05, as derived from previous studies on similar interventions (7, 8). A power of 0.8 and a significance level of 0.05 (two-tailed) were applied, yielding a required sample size of 64 participants per group. To assume an attrition rate at 20% and ensure robustness, we enrolled 80 participants per group (total *N* = 160). Every participant willingly took part in the study and provided their consent by signing an informed consent document. The study received ethical approval from the Ethics Committee of Haikou Hospital, which is affiliated with Xiangya School of Medicine, Central South University, under the reference number 2020-(Ethics Approval)-123.

The inclusion criteria for patients were as follows:

(1)  Patients who met the diagnostic criteria for stroke according to the Chinese classification of cerebrovascular diseases (2015) and were confirmed through brain computed tomography or magnetic resonance imaging (14).
(2)  Individuals experiencing their first episode of stroke between the ages of 18 and 70, with a disease duration of no more than 3 weeks.
(3)  Patients with a clear level of consciousness (alert, oriented to person, place, and time), no significant cognitive dysfunction or sensory aphasia, and the ability to follow simple commands and actively participate in examinations/treatments. Dysfunction primarily refers to motor impairments, though sensory impairments may also be present.
(4)  Patients with dysfunction in one limb (either arm or leg) or hemiparesis (dysfunction affecting one entire side of the body) were included.
(5)  Patients who provided informed consent by signing the required form.

The criteria for excluding patients were as follows:

(1)  Patients with dysfunction in both limbs.
(2)  Patients with severe pulmonary infections, cardiopulmonary failure, hepatic or renal insufficiency, and malignant tumors were excluded.
(3)  Patients with primary pulmonary dysfunction or respiratory issues caused by non-cerebrovascular conditions.

Drop-out criteria are listed below:

(1)  Patients who discontinued their participation in the experiments. Although no patients dropped out during the study, this criterion was included to account for potential discontinuation of participation.
(2)  Patients who could not endure electrotherapy.
(3)  Patients who were unable to collaborate with the treatment procedures.

The random number method was used to allocate patients into control and experimental groups, each consisting of 80 patients. Patients were randomly assigned to either the control or experimental group using a computer-generated random number table. Allocation concealment was ensured by using sealed opaque envelopes. Detailed demographic characteristics of the participants, including age, gender, disease duration, and lesion location, are presented in Table 1. No significant differences were observed between the groups at baseline (*p* > 0.05).

**Table 1:** Comparisons of gender, age, course of disease, lesion site, and body mass index of patients between the two groups.

Groups	Number of cases	Gender (number)		Age (years, x̄ ± s)	Hemiplegic side		Disease duration (days, x̄ ± s)	Body mass index (kg/m^2^)
		Male	Female		Right	Left		
Control group	80	51	29	59.91 ± 5.78	54	26	15.37 ± 2.84	23.12 ± 4.18
Experimental group	80	52	28	61.15 ± 5.24	50	30	16.13 ± 3.26	24.23 ± 3.98

The demographic data of the patients, including gender, age, disease duration, hemiplegic side, and body mass index, were analyzed in both groups, and the differences observed were not statistically significant (*p* > 0.05).

### 2.2 Treatment regimens

Patients in both cohorts received standard rehabilitation therapy, with the experimental group also undergoing a combination of electrical phrenic nerve stimulation and breathing exercises. Assessors and the physicians providing treatments were blinded.

**Figure 1:**
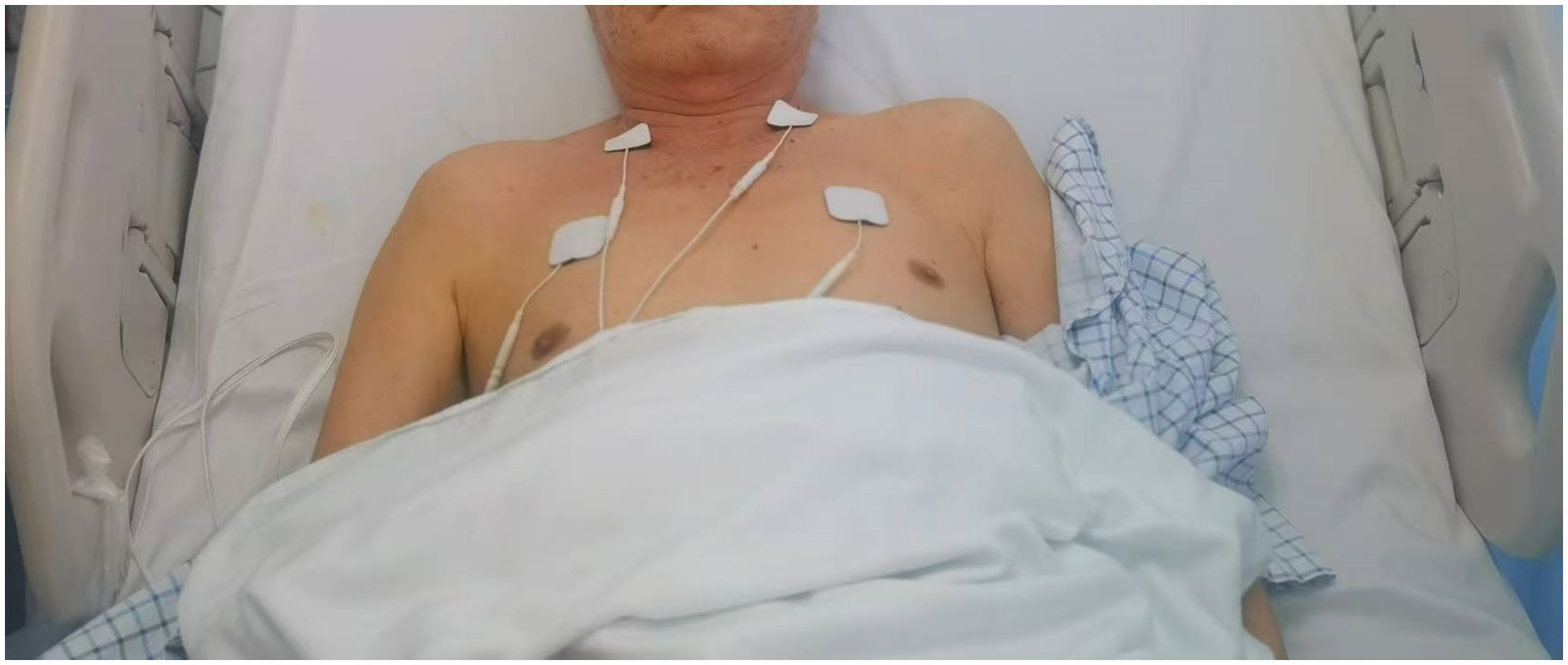
Extracorporeal diaphragm pacemaker and a representative setup on a subject.

(1)  The standard rehabilitation regimen included posture correction, passive joint mobility, Bobath therapy, balance drills, standing/walking training, and physical agents (e.g., electrotherapy). Sessions were divided into 2.5-h morning and afternoon blocks (total 5 h/day), with 10-min rest intervals every 50 min to minimize fatigue. Motor relearning techniques were integrated into functional tasks (e.g., transfers, walking).
(2)  Electrical stimulation of the phrenic nerve was carried out using an extracorporeal diaphragm pacemaker (HLO-GL13A; Guangzhou). This pacemaker had two sets of output electrodes, each comprising a small electrode (measuring 4 cm * 4 cm) and a large electrode (measuring 5 cm * 5 cm). To prepare for the procedure, the skin was cleaned with 75% alcohol cotton balls. The small electrodes were applied to the spot of the lateral border of the lower 1/3 of the sternocleidomastoid muscle, where the phrenic nerve runs along the surface of anterior scalene muscle, while the large electrodes were affixed to the surface of the pectoralis major muscle in the second intercostal space along the midclavicular line on the same side. The current intensity used during each session ranged from 10 to 30 mA, based on prior studies demonstrating safe and effective diaphragm activation (13). Electrodes were placed at anatomical landmarks for phrenic nerve targeting. Patients performed diaphragmatic breathing with abdominal pressure guidance, focusing on nasal inhalation and oral exhalation (Figure 1).
(3)  During the treatment, patients were positioned in a semi-reclining posture with their heads slightly tilted back to ensure optimal exposure of the sternocleidomastoid muscle. Bilateral electrical phrenic nerve stimulation was administered, starting with a low intensity and gradually increasing it, with a pacing frequency set between 8 to 12 times/min and an electrical stimulation frequency of 40 Hz. Bilateral stimulation was delivered sequentially (0.5-s delay between sides) to simulate physiological diaphragm activation. Synchronization was managed by the device’s dual-channel output. It was crucial that patients could tolerate the stimulation comfortably without experiencing significant pain. Patients remained still during the treatment and concentrated on monitoring their breathing patterns. This stimulation protocol was conducted five consecutive times per week, (20 min each time) for four consecutive weeks. Immediately following electrical phrenic nerve stimulation, patients were positioned supine, with relaxed hip and knee joints slightly flexed. They were then guided to breathe at a rate of 5–6 times/min, employing the technique of nasal inhalation followed by oral exhalation. Patients were instructed to place their left hand on their chest and their right hand on their abdomen. In cases of upper limb paralysis with sensory loss, therapists manually positioned the patient’s hands or used adaptive aids (e.g., weighted cuffs) to provide proprioceptive feedback. Visual guidance (e.g., mirrors) and verbal cues were integrated to compensate for sensory deficits during breathing exercises. To aid inhalation, gentle pressure was applied to the abdomen during abdominal expansion by rehabilitation therapist. At the conclusion of exhalation, the diaphragm was subjected to both vibration and stretching maneuvers. Diaphragm recordings were not taken, as the primary focus was on clinical outcomes such as pulmonary function and trunk control. This training regimen was administered to patients five consecutive times a week, (30 min each time) for four consecutive weeks (F igure 1). Adherence was monitored via session logs. No patients reported discomfort requiring discontinuation. Minor transient pain (<5% of sessions) resolved with intensity adjustment.

### 2.3 Efficacy evaluation

The trunk control and balance function of patients were assessed using the Sheikh Trunk Control Scale, Berg Balance Scale (BBS), and Balance Feedback Training Device (15) both before and after a four-week treatment period. Additionally, pulmonary function was measured using a pulmonary function instrument. All assessments were conducted by the same evaluator for consistency. All assessors received standardized training on the Sheikh Trunk Control Scale and Berg Balance Scale (BBS) and passed internal consistency tests to ensure scoring reliability.

(1)  Trunk control assessment was conducted using the Sheikh Trunk Control Scale, which comprised four bed-related movements: transitioning from lying on the back to the paralyzed side, transitioning from lying on the back to the unaffected side, sitting up from a supine position, and maintaining balance while sitting. These movements were assessed using a three-point grading system based on the quality of execution for each movement item: 0 points indicated an inability to perform the movement, 12 points indicated the ability to perform the movement but in an abnormal manner, and 25 points indicated the ability to complete the movement normally. The total score ranged from 0 to 100, with higher scores indicating better trunk control in the patient. Essentially, the Sheikh scale provides a means to gage a patient’s trunk control.
(2)  The BBS scale comprised 14 different movement tasks, such as transitioning from sitting to standing, standing to sitting, maintaining standing posture without support, closing eyes while standing, reaching forward with the upper arm, placing one foot alternately on a step, and balancing on one foot. Each task on the BBS was assessed using a 5-point scale, ranging from 0 to 4: A score of 0 meant the individual could not perform the task or needed significant assistance, while a score of 4 indicates the ability to perform the required action independently, and a score of less than 4 indicates the inability to perform the corresponding action independently. The total score on this scale ranged between 0 and 56, with higher scores reflecting better balance function.
(3)  The assessment of balance was conducted using a Balance Feedback Training Device known as the Pro-Kine Line-254P (PK-254P) from Tecnobody in Bergamo, Italy. This device was employed to examine the postural stability of patients. To evaluate stability while standing with eyes open, the PK-254P Balance Instrument was utilized in its static mode. The standard standing posture for patients consisted of the following criteria: (1) patients stood symmetrically along the A1-A5 axis, (2) they maintained a forward gaze with an upright head and raised chest, (3) the upper limbs rested naturally at their sides, (4) the medial edges of their feet were spaced 10 cm apart, and the highest points of their arches aligned with the A3-A5 axis. Postural stability was quantified via the Pro-Kine Line-254P device by analyzing movement length (total trajectory), movement area (sway area), and mean anterior–posterior/left–right movement speeds during static standing. The parameters under observation included movement length, movement area, as well as mean speeds of anterior–posterior and left–right movements.
(4)  Pulmonary function was evaluated, focusing on the assessment of inspiratory muscle function in patients using the POWERbreathe respiratory training device from POWERbreathe International Ltd. in Southam, UK. The patients were seated comfortably, and the training device was connected to a computer. To initiate inspiratory muscle training, the mouthpiece was inserted securely into the mouth, and patients followed cues to breathe in. Diaphragm muscle response was not directly monitored. Instead, the efficacy of the phrenic nerve stimulation was assessed through clinical outcomes, including improvements in pulmonary function (Maximum Inspiratory Pressure and Peak Inspiratory Flow) and trunk control (Sheikh Trunk Control Scale and Berg Balance Scale). These measures provide indirect evidence of diaphragm function and overall respiratory improvement.

Specifically, patients first exhaled as much air as possible from their lungs before taking a rapid and forceful breath to expand their chest. The gasses in their lungs were then gradually and passively expelled through the mouthpiece. Throughout this process, patients were observed to ensure that their chest and shoulder muscles remained relaxed.

The POWERbreathe training device has the capability to automatically assess inspiration by the patients. Baseline spirometry (e.g., FVC, FEV1) was performed pre-treatment to confirm no significant differences in pulmonary function between groups (*p* > 0.05). The assessment included the following parameters:

(1)  Maximum Inspiratory Pressure (MIP): This metric reflects the strength of inspiratory muscles during breathing.
(2)  Peak Inspiratory Flow (PIF): This measures the volume of inhaled gas per unit of time, indicating both breathing capacity and airway patency.

Each assessment was performed three times, and the results were averaged to obtain accurate measurements. Multiple tests, including the Sheikh Trunk Control Scale, Berg Balance Scale, and Balance Feedback Training Device, were used to comprehensively assess different aspects of balance ability, such as static and dynamic balance, as well as trunk control. Adverse events, including pain, dizziness, or skin irritation, were recorded daily. No severe complications occurred. Minor transient discomfort (e.g., skin redness) was reported in 5% of experimental group sessions but resolved without intervention.

### 2.4 Statistical analysis

SPSS 18.0 software was employed for the statistical analysis in this study, and *t*-tests were employed to compare measurement data. Specifically, the paired samples *t*-test was used for within-group comparisons, while the two independent samples *t*-test was applied for between-group comparisons. Differences were statistically significant at *p* < 0.05. All data are expressed as mean ± standard deviation.

## 3 Results

### 3.1 Comparisons of trunk function and balance function between the two groups before and after treatment

Before treatment, there were no notable statistical differences between the two groups in terms of Sheikh Trunk Control Scale scores (33.56 ± 11.05 in Control group vs. 36.35 ± 12.45 in Experimental group), BBS scores (35.56 ± 7.05 in Control group vs. 34.45 ± 6.55 in Experimental group), movement length (569.12 ± 165.35 mm in Control group vs. 571.56 ± 179.43 mm in Experimental group), movement area (754.25 ± 269.35 mm^2^ in Control group vs. 748.19 ± 276.96 mm^2^ in Experimental group), and mean anterior–posterior (15.83 ± 4.22 mm/s in Control group vs. 14.94 ± 4.35 mm/s in Experimental group) and left–right movement speeds (15.14 ± 4.38 mm/s in Control group vs. 14.65 ± 4.21 mm/s in Experimental group) (*p* > 0.05). However, following 4 weeks of treatment, there was a statistically significant improvement in Sheikh Trunk Control Scale (56.43 ± 12.23 in Control group, 76.78 ± 11.89 in Experimental group) and BBS scores (44.53 ± 6.23 in Control group, 69.26 ± 5.67 in Experimental group), along with a noticeable decrease in movement length (465.18 ± 122.54 mm in Control group, 438.86 ± 118.28 mm in Experimental group), movement area (628.04 ± 205.12 mm^2^ in Control group, 528.32 ± 179.14 mm^2^ in Experimental group), and mean anterior–posterior (12.28 ± 3.14 mm/s in Control group, 10.16 ± 3.25 mm/s in Experimental group) and left–right movement speeds (12.58 ± 3.23 mm/s in Control group, 9.95 ± 3.02 mm/s in Experimental group) (*p* < 0.05). The minimum clinically significant difference for the Sheikh Trunk Control Scale was 10 points, and for the Berg Balance Scale, it was 6 points. At the end of the four-week treatment period, statistically significant differences were observed in all measured parameters between the experimental and control groups (*p* < 0.05) (Tables 2, 3).

**Table 2:** Comparisons of Sheikh Trunk Control Scale scores and BBS between the two groups prior to and following treatment.

Groups	Number of cases	Time	Sheikh Trunk Control Scale score	BBS score
Control group	80	Before treatment	33.56 ± 11.05	35.56 ± 7.05
		After treatment	56.43 ± 12.23^a^	44.53 ± 6.23^a^
Experimental group	80	Before treatment	36.35 ± 12.45	34.45 ± 6.55
		After treatment	76.78 ± 11.89^ab^	69.26 ± 5.67^ab^

^a^ *p* < 0.05 compared within the group before treatment; ^b^ *p* < 0.05 compared with the control group.

**Table 3:** Comparisons of the distance traveled, spatial coverage, and average speed of movement in both groups prior to and following treatment.

Groups	Number of cases	Mean left–right movement speed (mm/s)	Mean anterior–posterior movement speed (mm/s)	Movement length (mm)	Movement area (mm^2^)
Control group					
Before treatment	80	15.14 ± 4.38	15.83 ± 4.22	569.12 ± 165.35	754.25 ± 269.35
After treatment	80	12.58 ± 3.23^a^	12.28 ± 3.14^a^	465.18 ± 122.54^a^	628.04 ± 205.12^a^
Experimental group					
Before treatment	80	14.65 ± 4.21	14.94 ± 4.35	571.56 ± 179.43	748.19 ± 276.96
After treatment	80	9.95 ± 3.02^ab^	10.16 ± 3.25^ab^	438.86 ± 118.28^ab^	528.32 ± 179.14^ab^

^a^ *p* < 0.05 compared within the group before treatment; ^b^ *p* < 0.05 compared with the control group.

### 3.2 Comparisons of pulmonary function between the two groups before and after treatment

Before treatment, there was no statistically significant difference in MIP (41.56 ± 8.76 cmH_2_O in Control group vs. 40.36 ± 9.68 cmH_2_O in Experimental group) and PIF scores (2.46 ± 0.15 L/s in Control group vs. 2.38 ± 0.16 L/s in Experimental group) between the two groups (*p* > 0.05). However, following 4 weeks of treatment, both groups demonstrated statistically significant improvements in their MIP (52.32 ± 9.04 cmH_2_O in Control group, 85.23 ± 10.42 cmH_2_O in Experimental group) and PIF scores (2.96 ± 0.17 L/s in Control group vs. 4.96 ± 0.18 L/s in Experimental group) (*p* < 0.05), and there was a statistically significant disparity between the two groups in these scores (*p* < 0.05) as shown in Table 4.

**Table 4:** Comparisons of inspiratory muscle function between the two groups prior to and following treatment.

Groups	Number of cases	MIP(cmH_2_O)	PIF(L/s)
Control group			
Before treatment	80	41.56 ± 8.76	2.46 ± 0.15
After treatment	80	52.32 ± 9.04^a^	2.96 ± 0.17^a^
Experimental group			
Before treatment	80	40.36 ± 9.68	2.38 ± 0.16
After treatment	80	85.23 ± 10.42^ab^	4.96 ± 0.18^ab^

^a^ *p* < 0.05 compared within the group before treatment; ^b^ *p* < 0.05 compared with the control group.

## 4 Discussion

Various causes can contribute to respiratory issues after a stroke. One of these causes is primary injury, which occurs when critical central respiratory routes in the brain, such as the brainstem and cerebral cortex, are damaged. Injuries to the respiratory centers in the brainstem, for example, may cause alterations in breath rate and rhythm (16). Damage to the coughing center can impair airway clearance, whereas cranial nerve injury can cause the glossopharyngeal muscle group to relax, resulting in glossoptosis and dysphagia. Furthermore, decreased reactivity of the chemoreceptors in the medulla oblongata to CO2 levels can cause either obstructive or central sleep apnea (16). The significant improvements in Maximum Inspiratory Pressure (MIP) and Peak Inspiratory Flow (PIF) in the experimental group suggest that the combination of electrical phrenic nerve stimulation and respiratory training effectively enhances respiratory muscle strength and airflow in early stroke patients. These findings are clinically relevant, as improved pulmonary function can reduce the risk of respiratory complications, such as pneumonia, and may contribute to shorter hospital stays.

The second factor to examine is the secondary damage mechanism, which is a common problem following a stroke (17). The experimental group demonstrated significant improvements in trunk control, as measured by the Sheikh Trunk Control Scale, and balance, as assessed by the Berg Balance Scale. These results indicate that the intervention not only targets respiratory function but also enhances core stability, which is critical for post-stroke recovery. Improved trunk control and balance may facilitate better mobility and reduce the risk of falls, which are common in stroke patients. Shoulder and hand pain can impair breathing, while excretory dysfunction causes gastrointestinal problems. As a result, the contraction and downward movement of the diaphragm are restricted, resulting in water-electrolyte imbalances and malnutrition. This aggravates diaphragmatic fatigue. Prolonged bed rest reduces skeletal muscle strength by 1 to 3% each day and causes the diaphragm to atrophy eight times quicker than skeletal muscle (18). This raises the likelihood of having difficulties clearing mucus and developing hypostatic pneumonia, which eventually leads to an increase in physiologic dead space.

The third factor to consider is iatrogenic injury. While mechanical ventilation is a quick and efficient way to establish respiratory support, it comes with the potential for complications such as ventilator-induced lung damage and diaphragmatic injury. Additionally, prolonged use of high-pressure ventilation can result in diaphragmatic weakening and impaired function (19). The phrenic nerve plays a crucial role in diaphragm function, which is essential for both respiration and postural control. It then travels down alongside the anterior scalene muscle, eventually entering the thoracic region between the subclavian artery and vein in order to reach the diaphragm (20). It is worth noting that phrenic nerve function can impact breathing by directly affecting the functioning of the diaphragm. The diaphragm, being the primary respiratory muscle, actively contracts to create negative pressure in the chest, providing 60 to 70% of the power for inhalation (7). Phrenic nerve stimulation likely enhances neuromuscular transmission and neuroplasticity in residual pathways, bypassing central deficits. Combined with respiratory training, it reinforces diaphragmatic-postural integration via viscero-somatic reflexes.

In a study using diaphragm ultrasound by Jung et al. (21), it was observed that in individuals with right hemiplegia, the mobility of the diaphragm was impaired on both sides, and there was a reduction in the strength and coordination of respiratory muscle groups. Khedr et al. (22) also reported a decrease in the amplitude of diaphragmatic movement on the affected side in 41% of stroke patients. Wang et al. (23) demonstrated that combining extracorporeal diaphragmatic pacing with respiratory feedback effectively reduced breathing difficulties, improved daily activities, and aided in removing tracheal tubes in post-stroke patients with tracheostomies.

Our study revealed that after 4 weeks of treatment, both the MIP and PIF values significantly increased compared to before treatment. Furthermore, these values were notably higher in the group receiving electrical phrenic nerve stimulation combined with breathing training compared to the control group. This outcome supports findings from a previous study (24). Mechanistically, it can be inferred that low-frequency electrical stimulation of the phrenic nerve influences the conduction of respiratory pathways, regulating the rhythm and strength of diaphragm movement to simulate normal respiratory patterns. Additionally, electrical phrenic nerve stimulation enhances the paralyzed side of the diaphragm in stroke patients, increasing its involvement in breathing, correcting abnormal breathing patterns, and expanding thoracic volume by improving diaphragm mobility (25). In stroke, cortical or brainstem lesions disrupt central respiratory drive, leading to diaphragmatic paresis. Electrical phrenic stimulation bypasses central deficits by directly activating the nerve, enhancing neuromuscular transmission and promoting neuroplasticity in residual pathways. Combined with respiratory training, it reinforces the diaphragm’s dual role in respiration and postural control via viscero-somatic integration.

Following a stroke, patients may experience a range of issues with their trunk muscles, leading to weakened control of their torso. This can result in problems with sitting, standing, and adopting abnormal postures like tilting the pelvis backward or flexing the trunk while walking or standing. The ability to control the trunk is closely linked to one’s balance. When the body faces external forces or changes, it needs to readjust itself, typically achieved through the quick contractions of various muscles including the rectus abdominis, obliquus internus abdominis, obliquus externus abdominis, trapezius muscle, latissimus dorsi, and erector spinalis muscle (6).

In our study, after 4 weeks of treatment, we observed significant improvements in both groups in terms of Sheikh Trunk Control Scale scores, BBS scores, movement length, movement area, and mean anterior–posterior and left–right movement speeds. Additionally, these measures were notably higher in the experimental group compared to the control group, aligning with prior research indicating that breathing training enhances post-stroke trunk and balance functions (26, 27). In conclusion, combining electrical phrenic nerve stimulation with breathing training can enhance trunk stability and improve balance function in stroke patients.

This study has several limitations, including the specific population and exclusion criteria, which may limit generalizability. Additionally, the short follow-up period and lack of detailed reporting on stimulation parameters suggest the need for further research. Future research should explore long-term outcomes and compare combined interventions with standalone therapies. Diaphragm function was inferred clinically; future work should include ultrasound or EMG for direct assessment.

In summary, the findings of this research revealed that the combination of electrical phrenic nerve stimulation and breathing exercises not only enhanced the respiratory function in early stroke patients but also improved their trunk control and balance.

## Data Availability

The raw data supporting the conclusions of this article will be made available by the authors, without undue reservation.
